# MicroRNA-154 functions as a tumor suppressor in osteosarcoma by targeting Wnt5a

**DOI:** 10.3892/or.2021.8130

**Published:** 2021-07-05

**Authors:** Hui Zhou, Minglei Zhang, Hongping Yuan, Wei Zheng, Chunyan Meng, Dongxu Zhao

Oncol Rep 35: 1851-1858, 2016; DOI: 10.3892/or.2015.4495

Following the publication of this paper, it was drawn to the Editors attention by a concerned reader that the western blotting data featured in Fig. 5B and C, and also in Fig. 6B, were strikingly similar to data appearing in different form in other articles by different authors at different research institutes. Owing to the fact that the contentious data in the above article were already under consideration for publication, or had already been published, elsewhere prior to its submission to *Oncology Reports*, the Editor has decided that this paper should be retracted from the Journal. After having been in contact with the authors, they agreed with the decision to retract the paper. The Editor apologizes to the readership for any inconvenience caused.

